# Exploring the application and challenges of fNIRS technology in early detection of Parkinson’s disease

**DOI:** 10.3389/fnagi.2024.1354147

**Published:** 2024-03-08

**Authors:** Pengsheng Hui, Yu Jiang, Jie Wang, Congxiao Wang, Yingqi Li, Boyan Fang, Hujun Wang, Yingpeng Wang, Shuyan Qie

**Affiliations:** ^1^Department of Rehabilitation, Beijing Rehabilitation Hospital, Capital Medical University, Beijing, China; ^2^Department of Critical Care Medicine, West China Hospital, Sichuan University, Chengdu, China; ^3^Department of Neurological Rehabilitation, Beijing Rehabilitation Hospital, Capital Medical University, Beijing, China

**Keywords:** Parkinson’s disease, functional near-infrared spectroscopy, machine learning, diagnostic model, application challenges

## Abstract

**Background:**

Parkinson’s disease (PD) is a prevalent neurodegenerative disorder that significantly benefits from early diagnosis for effective disease management and intervention. Despite advancements in medical technology, there remains a critical gap in the early and non-invasive detection of PD. Current diagnostic methods are often invasive, expensive, or late in identifying the disease, leading to missed opportunities for early intervention.

**Objective:**

The goal of this study is to explore the efficiency and accuracy of combining fNIRS technology with machine learning algorithms in diagnosing early-stage PD patients and to evaluate the feasibility of this approach in clinical practice.

**Methods:**

Using an ETG-4000 type near-infrared brain function imaging instrument, data was collected from 120 PD patients and 60 healthy controls. This cross-sectional study employed a multi-channel mode to monitor cerebral blood oxygen changes. The collected data were processed using a general linear model and β values were extracted. Subsequently, four types of machine learning models were developed for analysis: Support vector machine (SVM), K-nearest neighbors (K-NN), random forest (RF), and logistic regression (LR). Additionally, SHapley Additive exPlanations (SHAP) technology was applied to enhance model interpretability.

**Results:**

The SVM model demonstrated higher accuracy in differentiating between PD patients and control group (accuracy of 85%, f1 score of 0.85, and an area under the ROC curve of 0.95). SHAP analysis identified the four most contributory channels (CH) as CH01, CH04, CH05, and CH08.

**Conclusion:**

The model based on the SVM algorithm exhibited good diagnostic performance in the early detection of PD patients. Future early diagnosis of PD should focus on the Frontopolar Cortex (FPC) region.

## 1 Introduction

Parkinson’s disease (PD) is a prevalent neurodegenerative disorder characterized primarily by motor dysfunction, manifesting symptoms such as resting tremors, rigidity, bradykinesia, and postural instability (Mazzoni et al., 2012; Cheng and Su, 2020). As the second most common neurodegenerative condition in the elderly, the early diagnosis of PD holds paramount importance for timely intervention and improving patient quality of life (Aarsland et al., 2021). However, the early symptoms of PD can be confounded with other movement disorders such as Multiple System Atrophy, drug-induced Parkinsonism, and vascular Parkinsonism, making accurate early diagnosis a significant challenge (Tolosa et al., 2021). Currently, the diagnosis of PD heavily relies on clinical manifestations and the judgment of experienced clinicians, a method that may lack sensitivity and specificity, particularly in the early stages of the disease (Pahwa and Lyons, 2010; Postuma et al., 2015; Adler et al., 2021). An accurate and early diagnosis is crucial for paving the way for timely interventions, significantly enhancing the patient’s quality of life, and decelerating the progression of the disease (Welte et al., 2015).

With the rapid advancement of neuroimaging technologies, functional imaging has emerged as an essential tool for diagnosing and monitoring neurological disorders (Weiller et al., 2006). Functional near-infrared spectroscopy (fNIRS) stands out as a non-invasive, cost-effective, and user-friendly neuroimaging tool, showing potential in diagnosing and monitoring various neurological conditions (Sun et al., 2018). fNIRS monitors and records changes in cerebral blood oxygenation in real-time, reflecting the activity dynamics of cortical neurons. Its robust resistance to motion artifacts, coupled with superior temporal resolution compared to functional magnetic resonance imaging (fMRI) and better spatial resolution relative to electroencephalogram (EEG), positions fNIRS as a promising tool, particularly in identifying early cognitive impairments in PD patients (Oku and Sato, 2021; Pereira et al., 2023; Su et al., 2023). Current studies utilizing fNIRS have identified differences in frontal cortex activation in PD patients during motor tasks compared to healthy subjects (Feng et al., 2023).

Moreover, the expanding domain of artificial intelligence offers novel opportunities for employing fNIRS in diagnosing clinical disorders (Eastmond et al., 2022). Research indicates that machine learning algorithms can effectively differentiate various brain activities and emotional states based on fNIRS signals, suggesting the potential of this technology for early diagnosis and treatment monitoring of neurological diseases (Qiu et al., 2022a,b). Additionally, scholars have employed machine learning algorithms to unearth latent patterns and features in fNIRS data, developing a novel approach to understanding brain activity (Andreu-Perez et al., 2021; Oku and Sato, 2021; Eastmond et al., 2022). These studies contribute significantly to advancing neuroscience research and lay the groundwork for future clinical applications. However, to date, there has been a paucity of literature on constructing early diagnostic models for PD patients using fNIRS datasets.

This study pioneers the exploration of the feasibility of using fNIRS technology in conjunction with machine learning algorithms for the early diagnosis of Parkinson’s Disease. In this context, our research aims to evaluate the feasibility and effectiveness of integrating fNIRS technology with machine learning algorithms for the early diagnosis of PD. By doing so, we strive to fill a critical gap in the current diagnostic approach, leveraging the strengths of fNIRS in capturing cortical activation patterns and the analytical power of machine learning in deciphering complex data. This synergistic approach is anticipated to enhance the diagnostic accuracy for PD, especially in its early stages, thereby contributing significantly to the field of neurology and offering a beacon of hope for those afflicted by this debilitating condition.

## 2 Materials and methods

### 2.1 Study design and participant selection

This cross-sectional study involved 3 different groups of participants, PD-HY01 (Hoehn and Yahr Stage 1) group, PD-HY02 (Hoehn and Yahr Stage 2) group and control group. Detailed demographic and clinical characteristics of the participants are shown in Table 1.

**Table 1:** General characteristics of subjects.

	PD-HY01 group	PD-HY02 group	Control group
Number of people	60	60	60
Age (years)	54.93 ± 3.48	56.83 ± 3.22	57.80 ± 5.41
Male: female	27/33	35/25	31/29
Height (cm)	167.2 ± 4.2	168.2 ± 2.5	168.4 ± 5.4
Body weight (kg)	65.6 ± 3.8	64.8 ± 2.1	64.1 ± 8.3
Duration (weeks)	15.6 ± 5.2	20.9 ± 15.6	/
L-dopa equivalent doses (LEDs)	371.5 ± 159.5	379.1 ± 142.3	/

“/” means no value.

#### 2.1.1 Parkinson’s disease patients

In this study, the PD group consists of 120 patients (62 males and 58 females), all diagnosed with primary Parkinson’s disease by neurologists at the Department of Neurology, Beijing Rehabilitation Hospital, Capital Medical University. The diagnostic criteria employed were rigorously defined in accordance with the Movement Disorder Society Clinical Diagnostic Criteria for Parkinson’s Disease. The study utilized the modified Hoehn and Yahr (H&Y) staging system. Among these patients, sixty are in H&Y stage 1 (27 males, 33 females), and sixty are in H&Y stage 2 (35 males, 25 females). Inclusion criteria were as follows: (1) Newly diagnosed primary PD patients with no history of other diseases; (2) classified in stages 1–2 of the Hoehn and Yahr Scale; (3) Right-handed. Exclusion criteria included: (1) Secondary Parkinson’s syndromes; (2) History of cerebrovascular disease, neurosurgical operations, or brain tumors; (3) History of alcohol or drug dependence. Drop-out criteria were: (1) Occurrence of severe adverse events; (2) Failure to complete the testing according to the established protocol; (3) Voluntary withdrawal. Additionally, all patients were undergoing antiparkinsonian treatment during the study.

#### 2.1.2 Control group

The control group consisted of 60 staff members and outpatient check-up attendees from Beijing Rehabilitation Hospital, Capital Medical University, including 31 males and 29 females, age-matched with the PD groups. Exclusion criteria were: (1) Intracranial tumors, trauma, or other significant neurological disorders; (2) Major internal medical diseases; (3) Inability to complete the fNIRS examination.

### 2.2 Ethical approval and informed consent

This study was approved by the Ethics Committee of Beijing Rehabilitation Hospital, Capital Medical University (ethical approval number: 2022bkky-029). All participants provided written informed consent prior to their involvement in the study.

### 2.3 Data acquisition equipment

**Figure 1:**
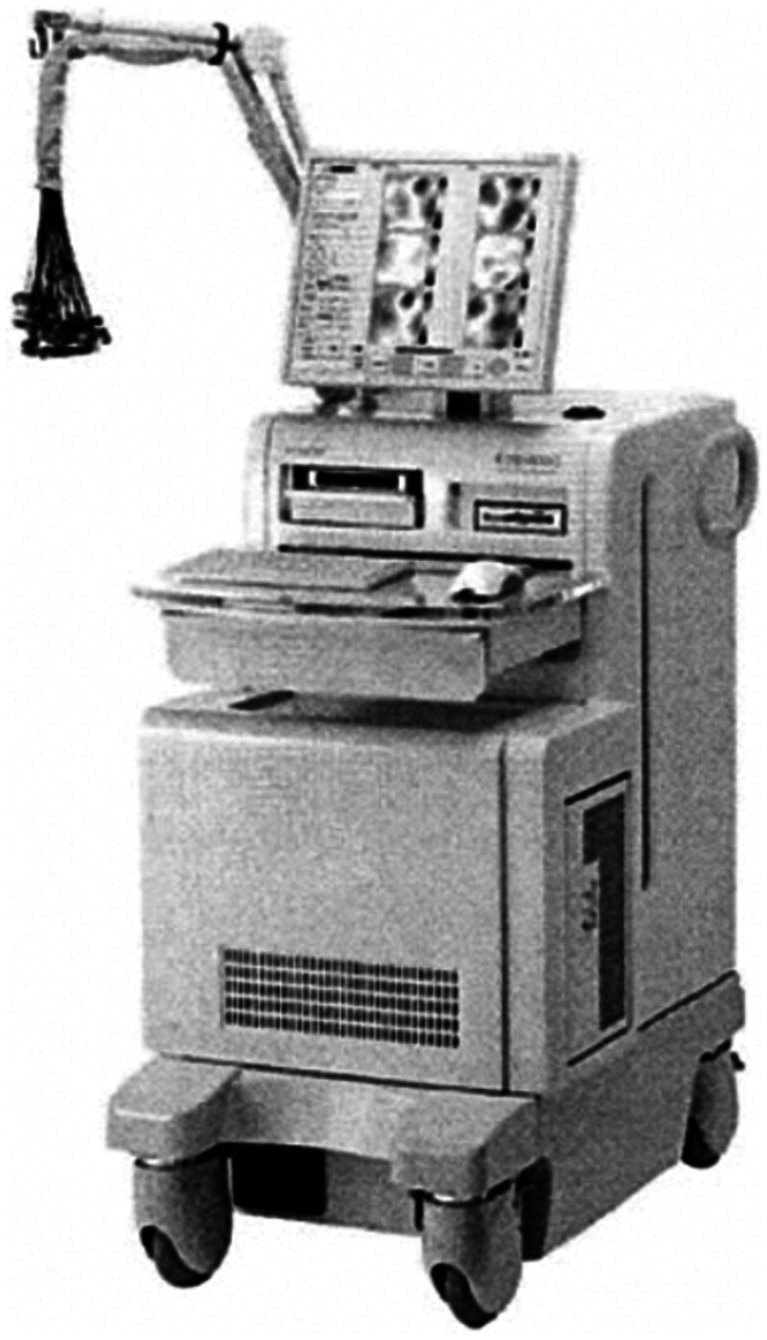
ETG-4000 device.

**Figure 2:**
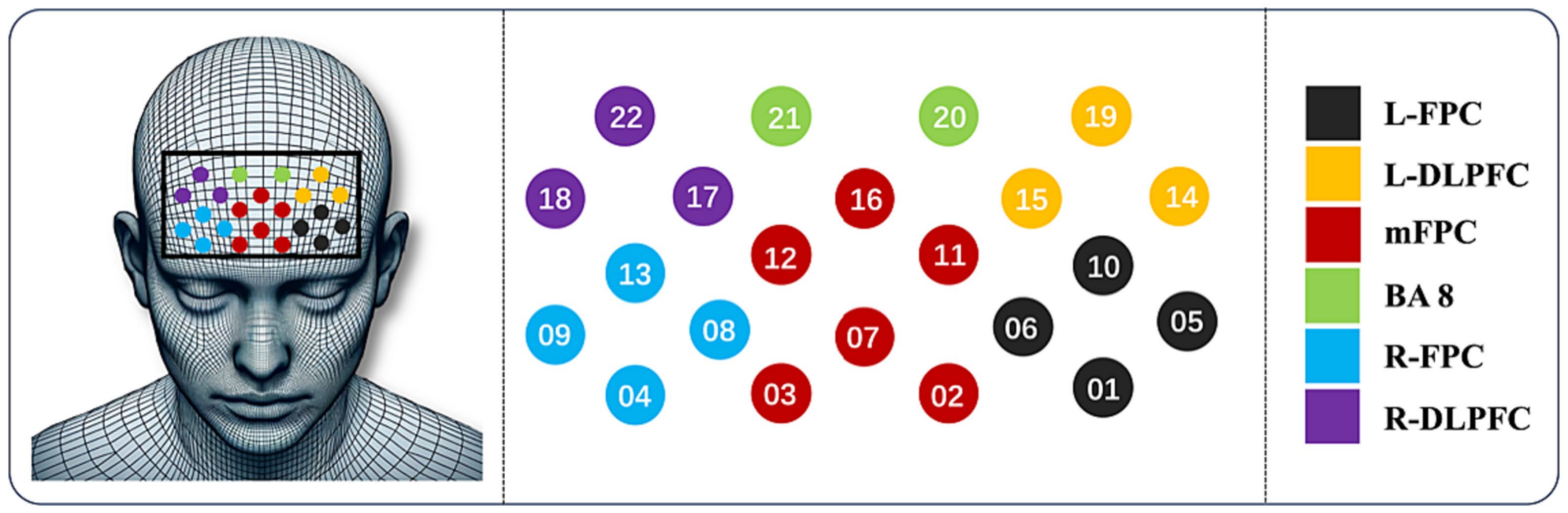
Optode and probe placement.

In this study, data was acquired using the ETG-4000 Optical Topography system, a fNIRS device, as shown in Figure 1. This equipment utilizes two wavelengths of near-infrared light (695 nm and 830 nm), delivered to the scalp through transmitting optical fibers and received by detecting fibers. The ETG-4000 can continuously measure changes in hemoglobin concentration in a multi-channel mode, calculating total hemoglobin concentration. In our experiment, we used an optode cap to measure the prefrontal cortex region of the participants. Customized for brain region specificity, the probe holder was equipped with 8 emitting and 7 detecting optodes (3 cm apart), forming 15 probes and 22 channels (CH). The channels are strategically distributed to cover significant cortical areas: CH01, CH05, CH06, CH10 for the Left-Frontopolar Cortex (L-FPC); CH04, CH08, CH09, CH13 for the Right-Frontopolar Cortex (R-FPC); CH02, CH03, CH07, CH11, CH12, CH16 for the medial Frontopolar Cortex (mFPC); CH14, CH15, CH19 for the Left-Dorsolateral Prefrontal Cortex (L-DLPFC); CH17, CH18, CH22 for the Right-Dorsolateral Prefrontal Cortex (R-DLPFC); and CH20, CH21 for Brodmann Area 8 (BA8). These regions play crucial roles in cognitive functions, decision-making, social cognition, complex problem-solving, and the integration of information across different brain regions. The optode holder securely fixes the transmitting and detecting optodes onto the scalp. The sampling frequency was set to 10 Hz, as depicted in Figure 2.

### 2.4 Experimental design and data collection

**Figure 3:**
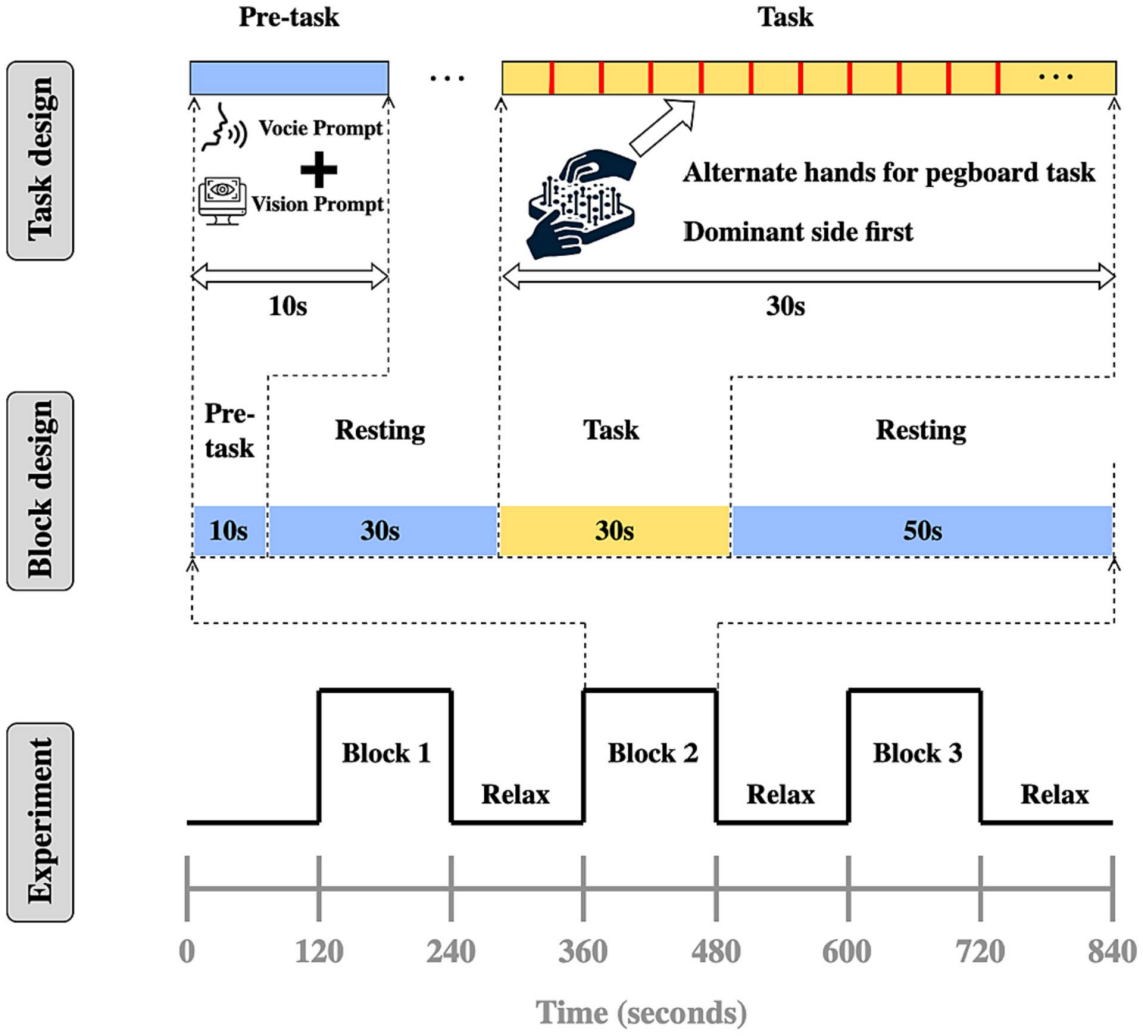
Experiment design.

**Figure 4:**
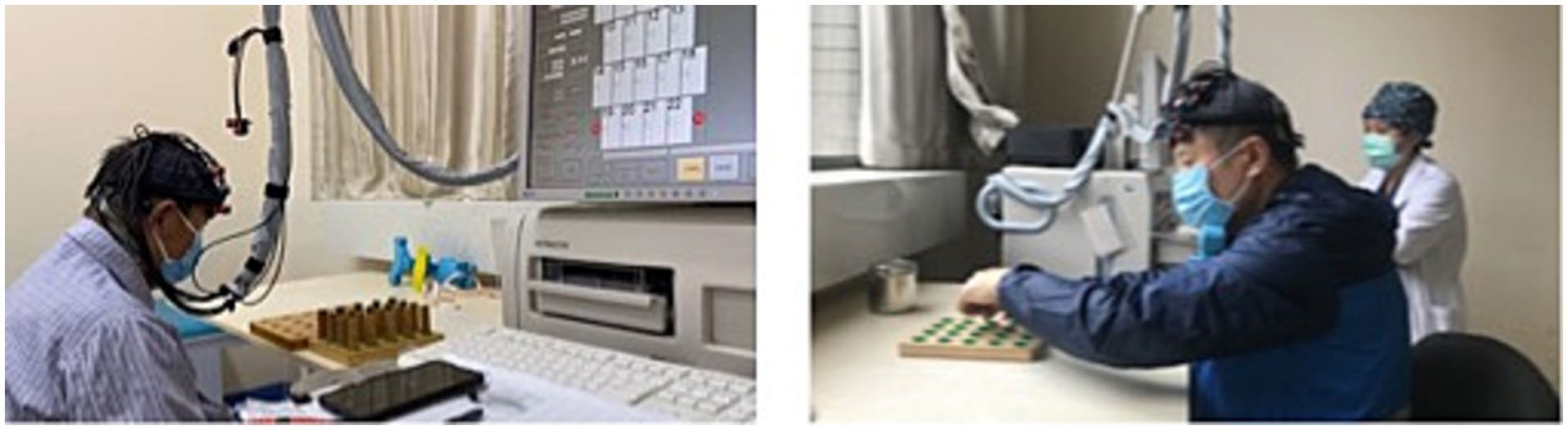
Histogram of data set distribution.

The experimental paradigm was based on a Block design, with each test cycle including a pre-task phase (10 s of blank screen), resting phase (30 s of blank screen), task phase (30 s of task execution), resting phase (50 s of blank screen), as illustrated in Figure 3. Data collection occurred in a quiet, light-controlled environment. Participants were asked to relax for 5 min before the experiment to minimize hemodynamic responses caused by prior activities. During the experiment, all potential environmental distractions were eliminated, and participants were instructed to remain relaxed, avoid unnecessary movement or thought, and sit comfortably in a chair, calming themselves before the start of the experiment. The ETG-4000 spectrometer was used to detect invalid channels, and the cap’s position and tightness were adjusted until the number of invalid channels was reduced to zero or one at most. During the task phase, subjects were required to use both hands continuously to complete a pegboard task. This task requires subjects to drive nails into the holes in the pegboard as quickly and accurately as possible, challenging their manual dexterity and coordination. According to recent research from institutions such as the University of Florida and Northwestern University, the pegboard task could provide objective, reliable data for tracking the progression of motor symptoms in Parkinson’s disease and atypical Parkinson’s disease (Wilkes et al., 2023). It is a practical, cost-effective measure that complements subjective clinical scales and expensive imaging techniques, providing a straightforward method for assessing efficacy in clinical trials and research. Auditory cues and system markers were used to delineate rest and task phases, as illustrated in Figure 4.

### 2.5 Data processing

To enhance the accuracy and validity of fNIRS test data, preprocessing is necessary. A low-pass filter with a cutoff frequency of 0.1 Hz is used to eliminate physiological noise such as cardiac noise, respiratory noise, and Mayer waves. The number of smoothing points is set to 5, and the Savitzky–Golay method is applied for data smoothing. The average intensity of hemoglobin signal changes in the 10 s before the start of the task is calculated for baseline correction. Channels with evident motion artifacts and poor signal quality are discarded prior to extracting hemodynamic data for analysis. In the test, linear regression is employed to fit the data of each channel during the stimulation phase to a horizontal line y = β, where β value reflects the level of channel activation. The β values of all 22 channels are used as inputs for the subsequent diagnostic models, forming a feature matrix of 180 × 22. Data processing and extraction are conducted using the NIRS_KIT software package (Hou et al., 2021) and Matlab (MathWorks, Natick, MA, United States, R2022b). Statistical analysis is performed using SPSS 26.0 statistical software. For data that is normally distributed and has homogeneous variances, independent sample t-tests are utilized for intergroup comparisons; for datasets not adhering to a normal distribution, we apply non-parametric tests, the Mann–Whitney U test, to ensure accurate statistical analysis. A significance level of *p* < 0.05 is set, indicating that differences are statistically significant.

### 2.6 Model building

The dataset matrix is combined with subject categories to form a 180 × 23 matrix, where the first 22 columns are used as inputs for the model, and the last column serves as the output value for model training and validation. Subsequently, the dataset undergoes standardization processes, including normalization, handling of outliers, management of missing values, and feature binarization. Data normalization was carried out using the Z-score normalization method, which involves subtracting the mean from each feature value and dividing by the standard deviation, ensuring that the data are on the same scale for easier model processing. Outliers were identified and handled using the Interquartile Range (IQR) method. For missing data, this study employed a multiple imputation approach to fill in missing values, based on the values of other variables, to maintain data integrity and minimize the bias that missing data might introduce. This study constructs four different diagnostic models to analyze and learn the task-state fNIRS data of PD patients: Support Vector Machine (SVM), Logistic Regression (LR), Random Forest (RF), and K-Nearest Neighbors (K-NN). The selection of machine learning models—SVM, K-NN, RF, LR—was strategic, aimed at leveraging their unique strengths for robust analysis. SVM was chosen for its proficiency in handling high-dimensional data, making it ideal for the complex fNIRS signals. K-NN’s simplicity and effectiveness in classification tasks complemented this approach, offering intuitive insights into data grouping. RF’s ensemble learning method was employed to mitigate overfitting risks, enhancing model generalizability. Lastly, LR was included for its transparent decision-making process, allowing straightforward interpretation of results. This multifaceted approach ensured a comprehensive analysis, underpinning our study’s methodological rigor. The hyperparameters for these four models are detailed in Table 2.

**Table 2:** Hyperparameters of each algorithm model.

Algorithm	Hyperparameters
LR	C: 10
	solver: liblinear
SVM	C: 10
	gamma: scale
	kernel: rbf
RF	n_estimators: 50
	max_depth: None
	min_samples_split: 10
K-NN	n_neighbors: 5
	weights: uniform
	algorithm: kd_tree

The study employs data splitting and cross-validation methods. The dataset is divided into a training set comprising 70% of the data and a validation set comprising 30%. The training set is used for model learning and tuning, while the validation set is used to assess the model’s performance and accuracy. To enhance the robustness and stability of the model evaluation, a 10-fold cross-validation method is applied. The training set is evenly divided into 10 subsets, and in each experiment, one subset is used as the validation set, while the remaining nine subsets are used for training the model. This process is repeated 10 times, giving each subset a chance to be used as the validation set. This method reduces the impact of randomness on model performance assessment, improving the stability and reliability of the results. Upon completion of the 10-fold cross-validation, the average of the 10 iterations was calculated, including accuracy, sensitivity, and specificity.

For a comprehensive evaluation of model performance, this study includes the calculation of confusion matrices and Receiver Operating Characteristic (ROC) curves. The confusion matrix provides detailed information about true positives, false positives, true negatives, and false negatives, aiding in understanding the model’s performance in differentiating between categories (Olivetti et al., 2015). The ROC curve, its “Area Under the Curve” (AUC), and the F1 score provide quantitative measures of a model’s overall performance and are vital tools for assessing classifier efficacy. These metrics are extensively utilized as comprehensive evaluation indicators in various diagnostic models. The ROC curve plots the true positive rate against the false positive rate at various threshold settings, enabling the visualization of a classifier’s performance across different thresholds. The AUC represents the degree to which the model can distinguish between classes; a higher AUC value indicates better model performance. The F1 score, a harmonic mean of precision and recall, is particularly useful in situations where an even balance between false positives and false negatives is critical. It is a single metric that combines the sensitivity and precision of the classifier, offering a balanced view of its performance, especially in cases of imbalanced datasets. These tools are integral in providing a holistic assessment of the classifier’s accuracy and reliability in diagnostic models.

### 2.7 Interpretability techniques

To enhance the interpretability of the model, particularly when dealing with black-box models, this study employs SHAP (SHapley Additive exPlanations) technology. SHAP is a method for explaining machine learning model predictions, aiding in understanding the contributions of different features to the model’s decision-making process and predictive outcomes (Stenwig et al., 2022). The core concept of SHAP is based on Shapley values from cooperative game theory, which decompose the influence of each feature into a degree of contribution to the prediction, thereby determining the importance of each feature for the final predictive outcome (Rodriguez-Perez and Bajorath, 2020). This approach enables the identification of features that have a positive or negative impact on the model’s output and their relative contribution, which is of significant value for further improvements to the diagnostic model (Park et al., 2021). The model construction process is completed using Python 3.11.

## 3 Results

### 3.1 Dataset distribution

**Figure 5:**
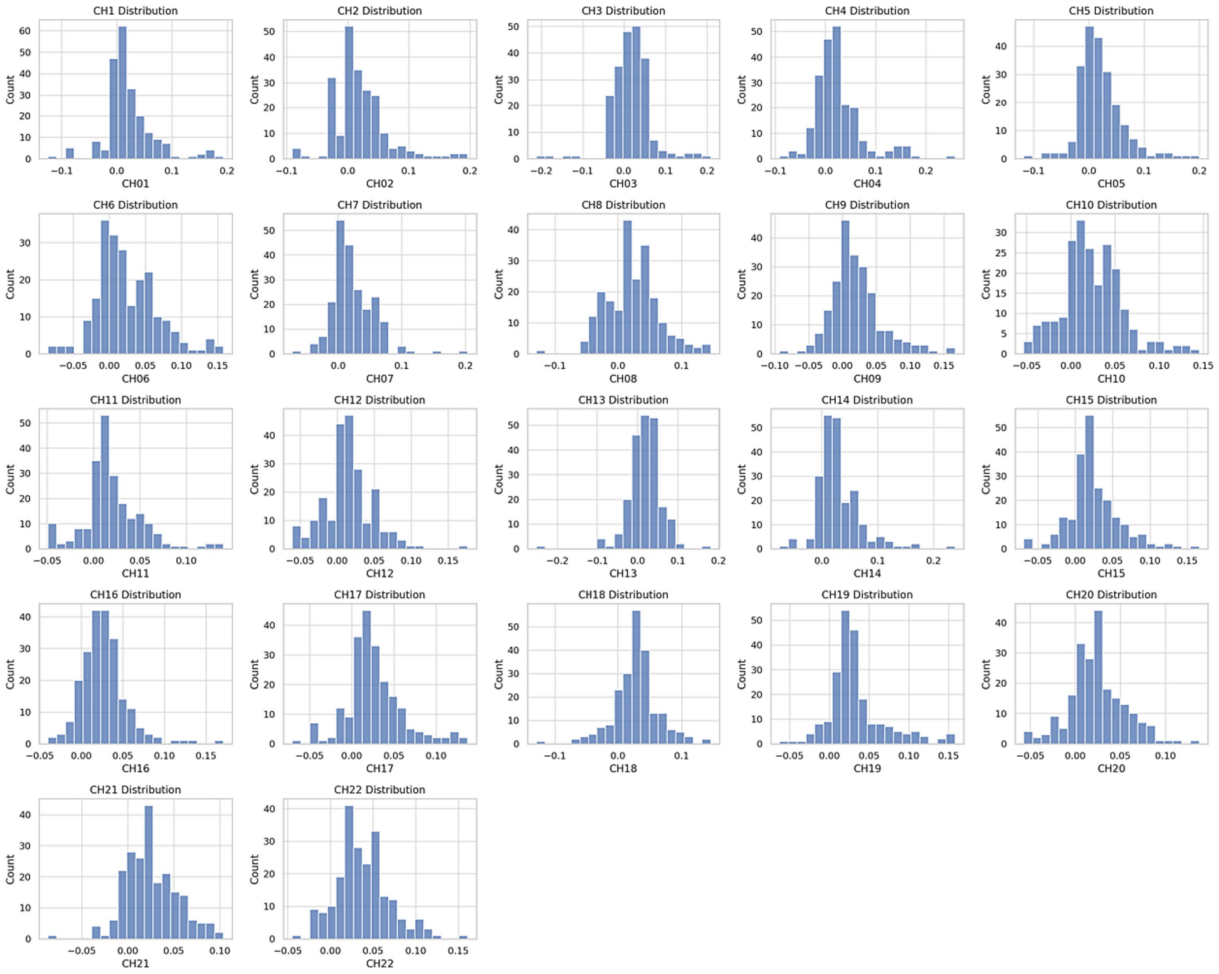
Participant testing procedure.

After preprocessing the dataset, a balanced distribution of data can be observed, as shown in Figure 5. The data within channels CH01 to CH22 exhibit uniformity and tend towards a normal distribution. This indicates that the distribution of attributes and labels within the dataset is relatively stable, without significant biases or imbalances.

### 3.2 Model predictions

**Figure 6:**
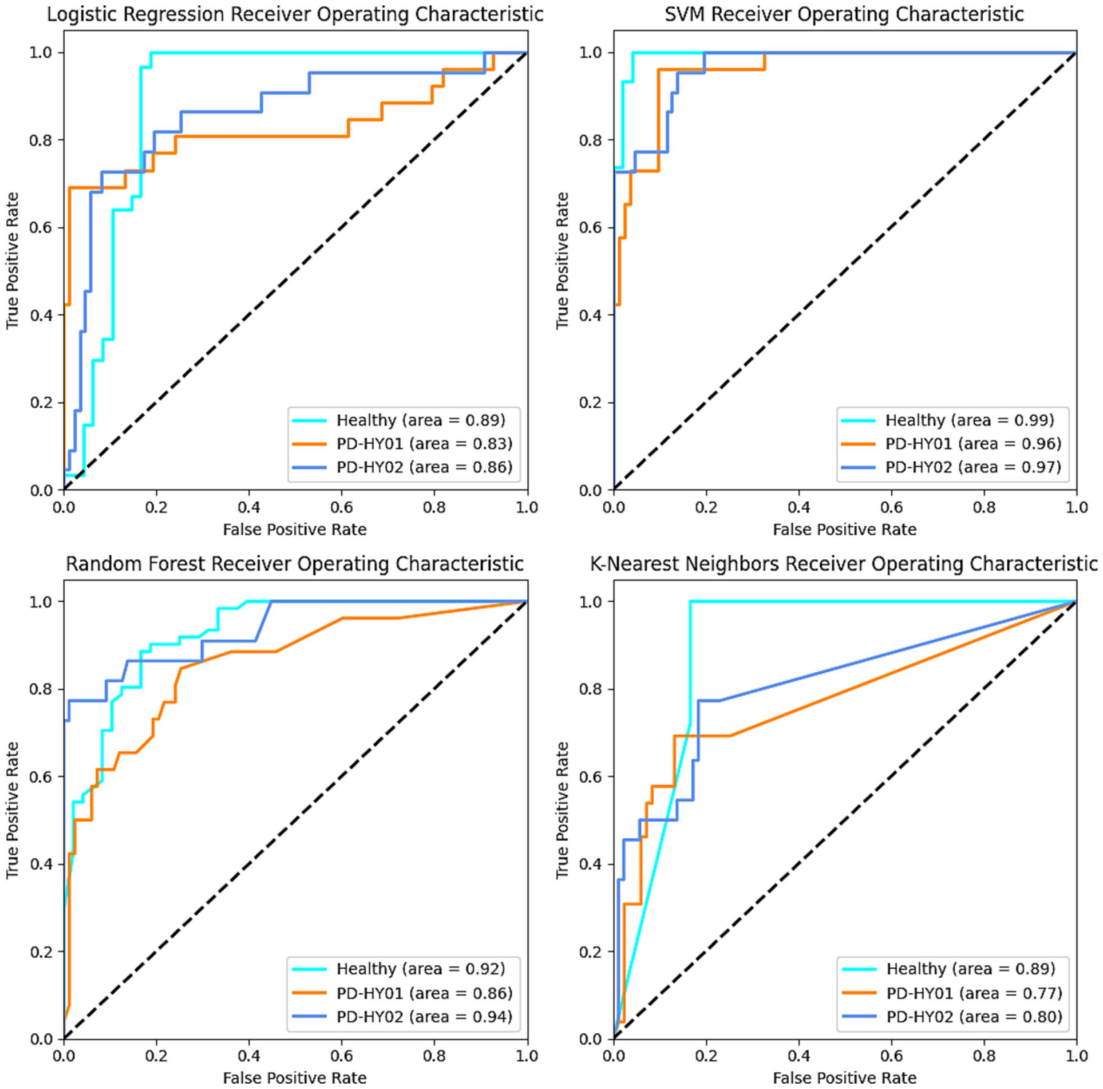
ROC results for each classifier curve.

**Figure 7:**
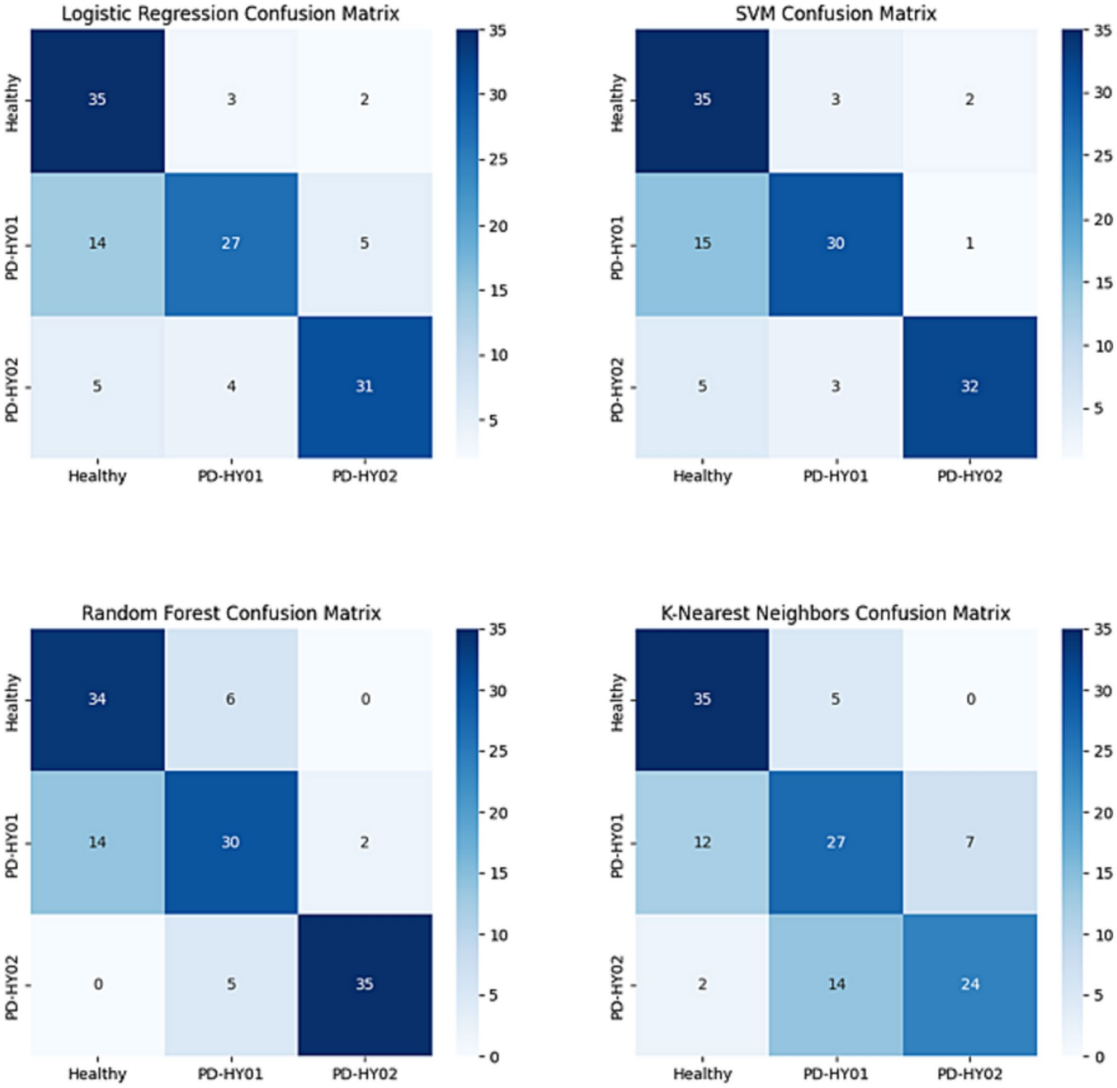
Confusion matrix results for each classifier.

The performance results of the four different predictive models are presented in Table 3. Overall, each model demonstrates certain capabilities in classifying fNIRS data, but the SVM algorithm shows superior overall performance, characterized by higher accuracy and reliability. Specifically, the SVM algorithm achieves an Accuracy of 85% and an F1 score of 0.85. Regarding the AUC, the best SVM model scores 0.99 for the control group, 0.96 for PD patients in H&Y stage 1, and 0.97 for those in H&Y stage 2, as illustrated in Figures 6, 7.

**Table 3:** Performance results of different classifiers.

Model	Accuracy	F1 Score	AUC
Logistic regression	0.82 ± 0.02	0.83 ± 0.03	0.83 ± 0.04
Support vector machine	0.85 ± 0.02	0.85 ± 0.01	0.95 ± 0.03
Random forest	0.79 ± 0.06	0.75 ± 0.05	0.89 ± 0.04
K-Nearest neighbors	0.73 ± 0.11	0.68 ± 0.12	0.81 ± 0.87

### 3.3 SHAP interpretability results

**Figure 8:**
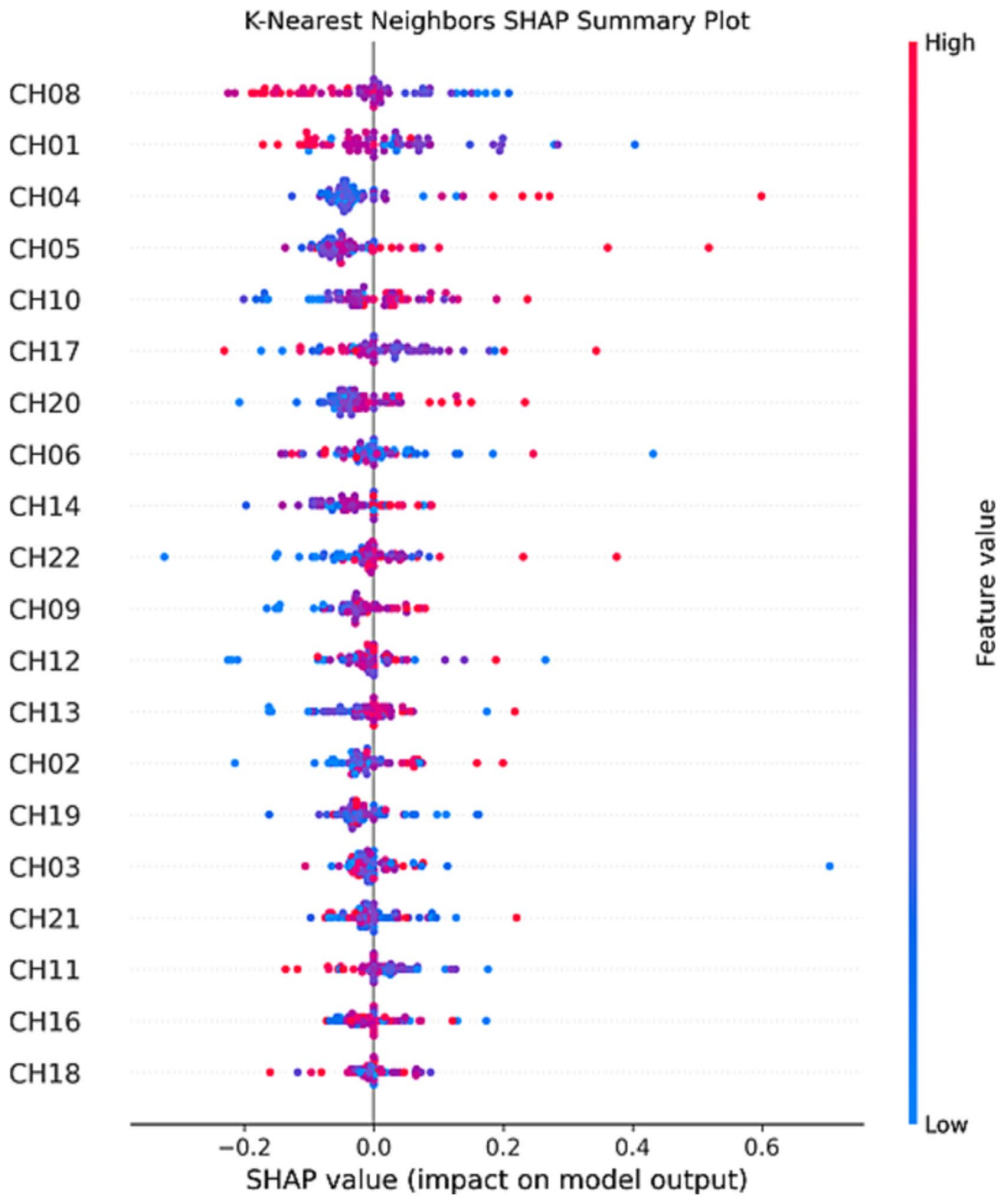
K-Nearest neighbors SHAP summary plot.

**Figure 9:**
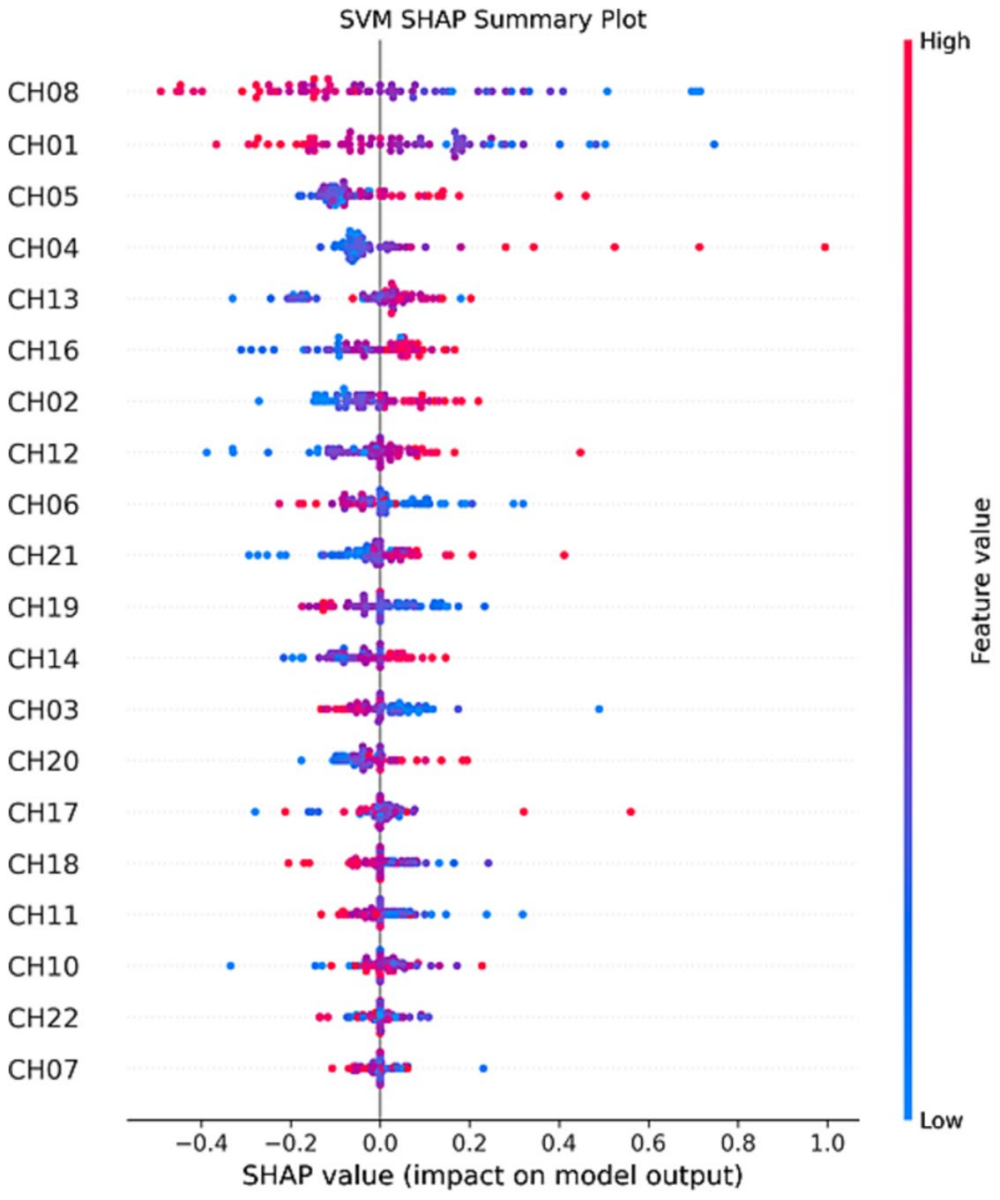
SVM SHAP summary plot.

**Figure 10:**
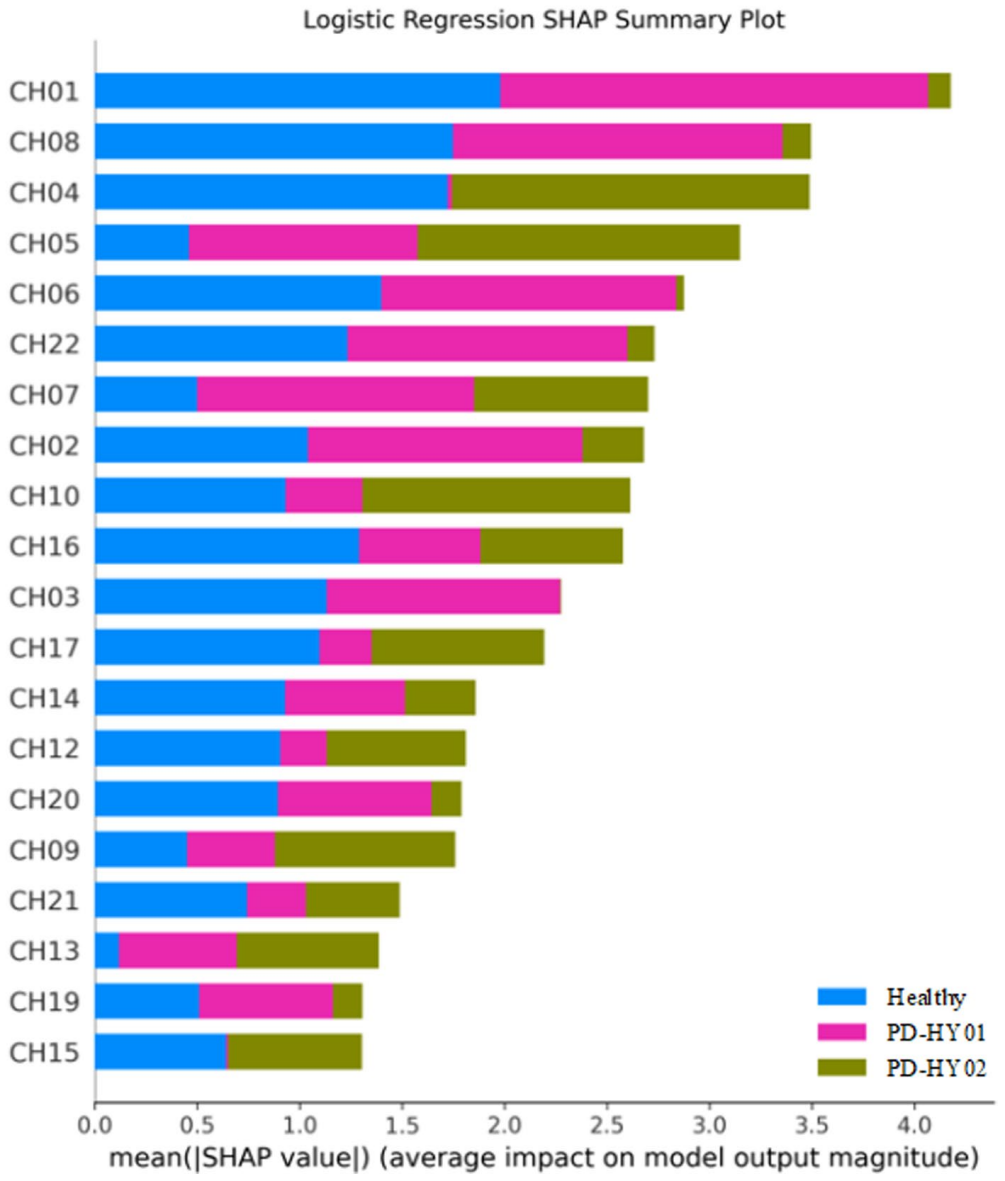
Logistic regression SHAP summary plot.

**Figure 11:**
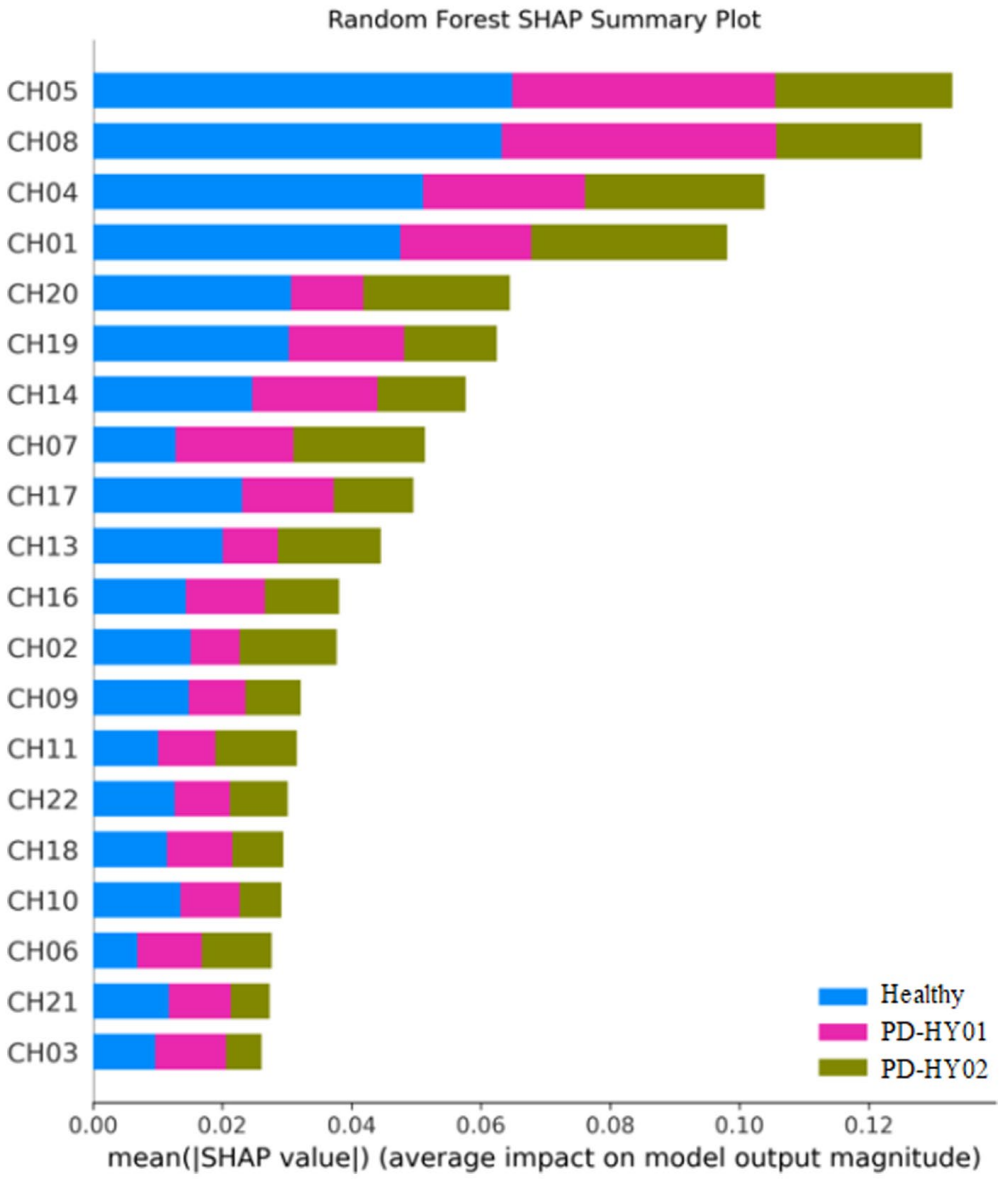
Random forest SHAP summary plot.

Interestingly, the application of SHAP technology for interpreting the four models reveals that channels CH01, CH04, CH05, and CH08 contribute most significantly to the model’s predictions, as visualized in Figures 8–11. These channels are located in the FPC region. This finding indicates that there is a difference in FPC activity between the two groups during task execution (specifically, a pegboard task using the dominant hand). This difference may suggest that the pattern of brain activity in PD patients during cognitive tasks is distinct from that of healthy participants.

## 4 Discussion

The early diagnosis of PD is paramount for effective patient management and prognosis, presenting a significant challenge within the medical diagnostic realm (Tolosa et al., 2021). Early detection not only significantly enhances disease management and treatment outcomes but also mitigates symptoms and decelerates disease progression (Pahwa and Lyons, 2010). PD is principally characterized by the progressive degeneration of neurons in the substantia nigra pars compacta, manifesting a range of motor and non-motor symptoms (Aarsland et al., 2021). The subtlety and lack of specificity of early symptoms often lead to the failure of traditional clinical diagnostic methods in accurately identifying PD at its onset (Adler et al., 2021). Presently, the diagnosis of early-stage PD heavily depends on medical observation and the assessment of clinical symptoms. However, these conventional approaches are susceptible to subjectivity, potentially culminating in misdiagnoses (Tolosa et al., 2021). The swift advancement of brain imaging technologies, including non-invasive techniques such as Positron Emission Tomography (PET), fMRI, and EEG, has led to their increased integration in detecting PD (Pagano et al., 2016). Concurrently, the evolution of artificial intelligence and pattern recognition technologies has rendered computer-assisted diagnostic tools indispensable in the early diagnosis of PD (Ripic et al., 2023). Despite the promise shown by the integration of advanced ML or DL algorithms with EEG signals, which exhibit marked differences in brain activation and functional connectivity between PD patients and healthy controls, several domains warrant further investigation to overcome the challenges of single-modality data reliance and the opaque nature of decision-making processes in current PD diagnostic models. Notably, most studies gather data with patients at rest, omitting motion or function-related data. This omission is significant as rehabilitation medicine, unlike clinical medicine, prioritizes functional impairments (Hudson, 2020). Moreover, the reliance on single-modality data in previous studies may limit a comprehensive understanding of task-state brain functional characteristics in PD patients (Makarious et al., 2022). Furthermore, the opacity of decision-making processes in current PD diagnostic models—the so-called “black box” effect—is notable. Although numerous ML and DL frameworks show promise in PD detection, a lack of model interpretability impedes understanding the diagnostic rationale, thereby hindering clinical application (Cruz et al., 2023).

In recent decades, the use of fNIRS in cognitive neuroscience has surged, benefiting from its advantages over other neuroimaging modalities like fMRI and EEG/MEG (Su et al., 2023). Notably, fNIRS is harmless, highly tolerant to physical movement, and extremely portable, making it suitable for all potential participant groups and experimental settings, both in and out of the laboratory (Grama et al., 2023). Exploring the combination of fNIRS with machine learning algorithms for early PD diagnosis represents a novel approach in the field of neurodegenerative disease diagnostics (Oku and Sato, 2021). In our study, the SVM model demonstrated excellent performance in differentiating PD patients from control group, with an accuracy of 85% and an F1 score of 0.85, highlighting its diagnostic accuracy. These results are consistent with previous studies, emphasizing the need for innovative non-invasive diagnostic methods for early PD detection (Krokidis et al., 2022). Moreover, SVM models have emerged as potent instruments in biomedical research, especially in classification and regression tasks involving high-dimensional data. The models’ capacity to identify the optimal hyperplane that maximizes the margin between classes in the feature space is crucial for precise prediction and classification in complex biomedical datasets (Cortes and Vapnik, 1995; Lundberg and Lee, 2017). Such a fundamental characteristic of SVMs facilitates the handling of the nuances in neuroimaging data, where distinguishing between healthy individuals and patients with neurological disorders, like PD, is often subtle and embedded within extensive datasets. The deployment of SVM models in neuroimaging data analysis has significantly propelled the field of disease diagnosis forward, offering a non-invasive and efficient means to early detect and differentiate neurological conditions. For example, neuroimaging techniques such as functional MRI and structural MRI produce voluminous data that encapsulates the functional and structural aspects of the brain. Analyzing this data with SVM enables the identification of patterns and biomarkers associated with diseases like PD, Alzheimer’s Disease, and schizophrenia, among others (Orrù et al., 2012; Steardo et al., 2020).

Within the PD context, SVM models have played a pivotal role in differentiating between PD patients and control group by analyzing fNIRS data for subtle changes imperceptible to the human eye. This capability is essential for the early diagnosis of PD, where timely intervention can significantly influence disease management and progression. Studies employing SVM models in conjunction with neuroimaging data have demonstrated high diagnostic accuracy and specificity, highlighting the models’ efficacy in biomedical applications (Buchlak et al., 2019). The SVM model’s high accuracy and F1 score further validate the effectiveness of machine learning methods in managing complex biomedical data.

One significant hurdle in utilizing machine learning models is their inherent “black box” nature, which obscures the decision-making process (Rudin, 2019). Addressing this, the implementation of interpretability techniques emerges as essential. Technologies such as SHAP play a pivotal role in demystifying the logic behind model predictions. Rooted in cooperative game theory, SHAP offers a comprehensive framework to elucidate any machine learning model’s output by assigning an importance value to each feature for a given prediction. This approach not only clarifies how predictive models function but also facilitates the discovery of biomarkers and critical attributes relevant to conditions like PD (Noble, 2006). Applying SHAP to SVM models, particularly in neuroimaging data analysis, represents a significant advancement towards unraveling the intricate biological and pathological phenomena underlying diseases. For instance, in PD diagnostics, SHAP values can identify brain regions and signals crucial for distinguishing PD patients from healthy controls, offering insights that not only improve model transparency but also guide further research and targeted therapeutic strategies (Molnar, 2020).

Specifically, during cognitive tasks, the notable influence of channels CH01, CH04, CH05, and CH08 in FPC indicates a deviation in brain activation patterns in PD patients relative to healthy individuals. This observation is instrumental in dissecting the neural mechanisms of PD, potentially shaping the development of precise therapies or interventions. The FPC’s integral role in high-level cognitive functions, such as decision-making, problem-solving, and social cognition, underscores its significance in complex cognitive processes, rendering it a vital focus for neurodegenerative disease research. Its strategic relevance is amplified by its connectivity with diverse brain networks, facilitating the integration of cognitive and emotional data to influence behavior and decision-making (Gilbert et al., 2006; Burgess et al., 2007). Concentrating on the FPC might shed light on the early cognitive and neural alterations linked to PD, extending the focus beyond conventional motor symptoms. Prior studies have emphasized the FPC’s role in cognitive functionalities and its potential alterations due to PD pathology (Goldman et al., 2018; Aarsland et al., 2021). Investigating the FPC’s role in executive functions could reveal how PD impacts brain regions tasked with high-order cognitive processes, significantly enriching our understanding of the disease’s progression and its impact on patient quality of life (Daffner, 2010). The emphasis on the FPC in future research is warranted not merely due to its pivotal role in cognitive functions and decision-making but also for its potential to deepen our understanding of PD. Such targeted research promises to broaden diagnostic, therapeutic, and rehabilitative approaches, significantly refining PD management strategies. By exploring the FPC’s involvement more thoroughly, we can discover new avenues for early diagnosis, personalized medicine, and targeted interventions, ultimately enhancing PD patients’ prognosis and quality of life. Moreover, network analyses employing resting-state functional MRI (rs-fMRI) are increasingly utilized in PD patient studies to identify and substantiate neurodegenerative disease associations (Albano et al., 2022). These networks serve not only as markers for disease processes but also as supplementary tools for clinical diagnosis and therapeutic trial screenings (Filippi et al., 2019). A study that applied SHAP in interpreting SVM-based neuroimaging analysis for PD underscored the FPC’s substantial role, offering a profound insight into the disease’s neuroanatomical foundations. Such interpretability is vital for bridging the gap between machine learning predictions and clinical decision-making, enabling a more informed and nuanced approach to disease diagnosis and management (Rodriguez-Perez and Bajorath, 2020).

Despite its advantages, the application of SHAP in enhancing the interpretability of SVM models in biomedical research is not devoid of challenges. The computational complexity of calculating SHAP values, especially for large datasets common in neuroimaging studies, poses a significant hurdle. Additionally, while SHAP provides a more intuitive understanding of model predictions, translating these insights into actionable clinical strategies requires careful consideration and further validation. Future research should focus on developing more efficient algorithms for computing SHAP values and exploring methods to integrate these interpretations into clinical workflows seamlessly. Moreover, the potential of SHAP to uncover novel biomarkers and therapeutic targets warrants further exploration, with interdisciplinary collaboration between computer scientists, biologists, and clinicians being pivotal for leveraging these insights to improve patient care.

Despite encouraging results, early PD diagnosis remains a complex and evolving field. The non-specific nature of early PD symptoms and the lack of reliable biomarkers contribute to this complexity (Ma et al., 2023). This study highlights fNIRS’s potential in identifying distinct cerebral blood flow patterns between PD patients and control group, underlining its promise as an objective indicator for early PD diagnosis. Yet, it’s crucial to clarify that our findings primarily suggest the potential utility of fNIRS, rather than definitively establishing its diagnostic capability. Further research is necessary to validate fNIRS as a reliable diagnostic tool for PD, emphasizing the need for integrating it with other diagnostic modalities and exploring larger, more diverse datasets. We must also acknowledge limitations in our study, such as potential biases in sample selection and unconsidered variables like lifestyle and genetic factors that may affect fNIRS data and diagnostic accuracy. Future research should focus on refining these diagnostic procedures, considering a wider range of machine learning models and larger datasets to improve the accuracy and reliability of early PD diagnosis.

## 5 Conclusion

In conclusion, this study paves the way for future research to explore more comprehensive machine learning models and integrate larger, more diverse datasets. Advances in neuroimaging and machine learning hold great promise for improving early PD diagnosis, potentially leading to better patient outcomes and more effective management strategies. Future research must also address the potential biases and unconsidered variables identified in this study to develop more robust diagnostic models.

## Data availability statement

The raw data supporting the conclusions of this article will be made available by the authors, without undue reservation.

## Ethics statement

The studies involving humans were approved by Ethics Committee of Beijing Rehabilitation Hospital. The studies were conducted in accordance with the local legislation and institutional requirements. The participants provided their written informed consent to participate in this study. Written informed consent was obtained from the individual(s) for the publication of any identifiable images or data included in this article.

## Author contributions

PH: Data curation, Formal analysis, Methodology, Project administration, Resources, Software, Visualization, Writing – original draft. YJ: Data curation, Investigation, Project administration, Resources, Software, Writing – original draft, Writing – review & editing. JW: Writing – original draft, Data curation. CW: Writing – original draft, Project administration, Methodology, Formal analysis, Data curation. YL: Writing – review & editing, Software, Methodology, Formal analysis. BF: Data curation, Formal analysis, Methodology, Resources, Supervision, Writing – original draft. HW: Formal analysis, Investigation, Methodology, Writing – original draft. YW: Writing – review & editing, Writing – original draft, Validation, Supervision, Software, Investigation, Conceptualization. SQ: Writing – review & editing, Writing – original draft, Visualization, Software, Resources, Formal analysis, Data curation, Conceptualization.
